# Toxicokinetic and Partial Mass Balance Assessment of ^14^C-Alpha Olefins in Rats

**DOI:** 10.3390/jox16010026

**Published:** 2026-02-02

**Authors:** Quan Shi, Jamie Dunn, Juan-Carlos Carrillo, Michael G. Penman, Robert H. Powrie, Corinne Haines, Hua Shen, Yuan Tian, Sophie Jia, Fabienne Hubert, Peter J. Boogaard

**Affiliations:** 1 Shell Global Solutions International B.V., Carel van Bylandtlaan 16, 2596 HR The Hague, The Netherlands; 2 Penman Consulting BVBA, Avenue des Arts 10, 1210 Brussels, Belgium; jamie.dunn@penmanconsulting.com (J.D.);; 3 CXR Biosciences Ltd., 2 James Lindsay Place, Dundee Technopole, Dundee DD1 5JJ, UK; 4 Shell Oil Company, 150 N. Dairy Ashford Rd., Houston, TX 77079, USA; 5 Faculty of Brain Sciences, University College London, 11-43 Bath St., London EC1V 9EL, UK; 6 Chevron Phillips Chemical Company, 9500 Lakeside Blvd., Houston, TX 77381, USA; 7 INEOS Oligomers, Hawkslease, Chapel Lane, Hampshire SO43 7FG, UK; fabienne.hubert@ineos.com; 8 Division of Toxicology, Wageningen University and Research, Stippeneng 4, 6708 WE Wageningen, The Netherlands; peter.boogaard@wur.nl

**Keywords:** toxicokinetic, disposition, mass balance, higher olefins, alkene, oral exposure

## Abstract

Higher olefins are a class of alkenes widely used as intermediates in the production of essential consumer and industrial products. This radiolabel disposition and partial mass balance study investigated the distribution and excretion of four ^14^C-radiolabelled alpha higher olefins (i.e., 1-octene, 1-decene, 1-hexadecene, and 1-eicosene) in male Wistar rats following a single oral dose (100 mg/kg). Blood, liver, kidney, adipose tissue, urine, and faeces were collected and analysed for total ^14^C-derived radioactivity. Urinary elimination was rapid, with approximately 70% and 90% of total radioactivity recovered in urinary excreted within 24 and 48 h, respectively. Excretion patterns showed a clear chain-length-dependent trend: shorter-chain olefins (C8, C10) exhibited higher urinary excretion, indicating greater systemic absorption, while longer-chain olefins (C16, C20) were primarily eliminated via faeces, suggesting limited intestinal uptake. Tissue distribution was minimal in blood, liver, and kidney, but adipose tissue retention increased with chain length. Total recovery of administered radioactivity in the analysed matrices was low, ranging from 17% to 60%. Importantly, because exhaled ^14^CO_2_ and volatile parent compounds were not captured, the missing fraction cannot be quantified and the balance cannot be considered closed. All in all, the current study describes the partial disposition of higher olefins and highlights the influence of molecular size and lipophilicity on the biological fat, though further studies are required to fully characterise their metabolic profile and total elimination kinetics.

## 1. Introduction

Higher olefins are a group of alkenes characterised by a single double bond between adjacent carbon atoms, with carbon chain lengths ranging from C6 to C54 [1]. These compounds serve as important intermediates in the production of various chemical products, including lubricants, surfactants, agricultural chemicals, alcohols, and plasticisers [2]. Industrially, higher olefins are typically produced through ethylene oligomerisation or olefin metathesis [3]. Based on the position of the double bond and the molecular structure, higher olefins can be classified into four categories: (1) linear alpha olefins (straight-chain alkenes with a double bond at the alpha position); (2) linear internal olefins (straight-chain alkenes with an internal double bond); (3) branched alpha olefins (branched alkenes with an alpha-position double bond); and (4) branched internal olefins (branched alkenes with an internal double bond).

Generally, higher olefins exhibit low systemic toxicity, with no-observed-adverse-effect levels (NOAELs) typically at or above 500 mg/kg body weight/day [4,5]. However, for the purposes of regulatory evaluation and the characterisation of safety profile, it is essential to understand its toxicokinetic (TK) properties including absorption, distribution, metabolism, and excretion (ADME), which provide critical insights linking external exposure to observed toxicological effects [6].

Previous studies using rat everted gut sacs have shown that higher olefins with chain lengths from C6 to C10 are readily absorbed, whereas those longer than C14 are poorly or not absorbed [1]. Metabolism of higher olefins primarily occurs in the liver, where cytochrome P450 enzymes initially convert them to transient epoxides, which are subsequently hydrolysed to glycols or conjugated with glutathione [7,8,9,10,11,12,13]. These intermediates are further metabolised and excreted in the urine as mercapturic acids [14]. Notably, linear alpha olefins are metabolised more efficiently than internal or branched olefins due to reduced steric hindrance near the double bond [7,11].

Distribution studies have shown that, following inhalation, linear alpha olefins (C8, C9, C10) are more readily absorbed and retained in rat tissues with the highest concentrations found in adipose tissue [15]. Additionally, tissue accumulation appears to increase with chain length. However, data regarding the distribution and excretion of linear alpha olefins following oral exposure remain limited. To address this data gap, the present study investigates the biodistribution and excretion profiles of higher olefins following a single oral administration. Mass balance studies are widely recognised as a comprehensive method to assess the fate and routes of elimination of chemicals in vivo [16,17]. Radiolabelled compounds, particularly those labelled with carbon-14 (^14^C), are considered the gold standard for quantifying chemical distribution in biological matrices such as plasma, urine, and faeces [18]. Accordingly, a study using ^14^C-labelled linear alpha olefins was conducted to characterise their in vivo distribution and excretion. However, this work is specifically designed as a partial mass balance study. The scope is focused on the systematic generation of quantitative data within primary biological matrices (e.g., plasma, urine, faeces, and selected tissues) to assess bioaccumulation potential and terminal excretion routes. Importantly, this study does not include the experimental collection of exhaled air or CO_2_ trapping as key methodological limitation; as such, the results represent a targeted evaluation of non-volatile residues rather than a complete total mass balance, and therefore the study does not permit calculation of a closed or complete mass balance. This work provides an incremental but essential contribution to the applied toxicology and regulatory registration of linear alpha olefins by characterising the influence of chain length and lipophilicity on their disposition in vivo.

## 2. Materials and Methods

### 2.1. Test Items and Chemicals

**Figure 1:**
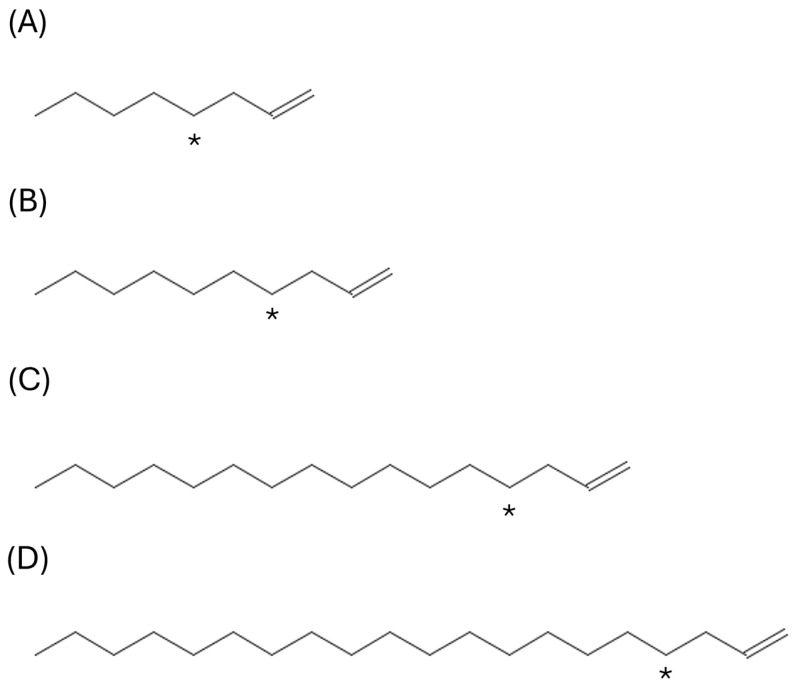
Chemical structure of (**A**) 1-Octene, (**B**) 1-Decene, (**C**) 1-Hexadecene, and (**D**) 1-Eicosene. * denotes the site of radiolabelled carbon.

Four unlabelled alpha olefins: 1-Octene (CAS No. 111-66-0), 1-Decene (CAS No. 872-05-9), 1-Hexadecene (CAS No. 629-73-2) and 1-Eicosene (CAS No. 3452-07-1) were supplied by the Higher Olefins and Poly Alpha Olefins (HOPA) REACH consortium. The carbon-14 (^14^C) labelled alpha-olefins (carbon position 4) (Figure 1) 1-Octene, 1-Decene, 1-Hexadecene and 1-Eicosene were supplied by Blychem Ltd., Billingham, UK. All ^14^C-labelled alpha-olefins were supplied with specific activities of 10 mCi/mmole. Handling of these materials were in accordance with the United Kingdom Control of Substances Hazardous to Health (COSHH) Regulations, and the normal safety precautions as detailed in the relevant CXR Biosciences Ltd. (Dundee, UK). Standard Operating Procedures (SOP).

### 2.2. Animals

The animal studies were conducted by CXR Biosciences Ltd. (Dundee, UK). in the Medical School Resource Unit (MSRU), Dundee University (Dundee, UK) using male Han Wistar rats obtained from Harlan, Bicester, UK. The rats (approx. 8–10 weeks old on arrival) were acclimated for a period of at least 5 days and examined to ensure their health status prior to dosing. The animal room environment was controlled to maintain a temperature of 19–23 °C, relative humidity within a range of 40–70%, nominal 14–15 air changes per hour, and a 12 h light/12 h dark cycle, suitable for the Han Wistar strain of rat. Drinking water (local supply in bottles) and RM1 powdered diet (Special Diet Services Ltd., Stepfield, Witham, Essex, UK) were available to animals ad libitum prior to and throughout the study.

The in vivo procedures undertaken during the course of this study were subject to the provisions of the United Kingdom Animals (Scientific Procedures) Act 1986. The Act, administered by the UK Home Office, regulates all scientific procedures in living animals which may cause pain, suffering, distress or lasting harm and provides for the designation of establishments where procedures may be undertaken, the licensing of trained individuals who perform the practical techniques, and the issue of project licences for specified programmes of work. This study complied with all applicable sections of the Act and the associated Codes of Practice for the Housing and Care of Animals used in Scientific Procedures and the Humane Killing of Animals under Schedule 1 to the Act, issued under Section 21 of the Act.

### 2.3. Dose Formulation

The dosing solution of each alpha olefin was formulated in corn oil, the preferred vehicle for complete homogeneous solubility [4,5,19], containing 190 µL of unlabelled alpha olefins, 10 µL of ^14^C-alpha olefins, and 19.8 mL of corn oil to generate an oral formulation with a volume of 20 mL and 10 μCi/100 mg on the day before dosing.

The total radioactivity dosed to each animal (Table 1) was performed on 50 µL of the dosing solutions of each alpha olefins and the total amount of radioactivity administered to each animal was calculated using the volume of dosing solution administered by oral gavage.

### 2.4. Study Design

In total 16 rats were uniquely numbered by ear punch and allocated into four groups (n = 4 per group, sufficient for testing [20]) so that the groups were approximately weight-matched up to one week after arrival. Within each group, four male rats were administered a single oral gavage of [^14^C]-alpha olefins at 100 mg/kg bodyweight with a dosing volume of 10 mL/kg bodyweight. Four rats were housed individually in glass metabolism cages 24 h prior to dosing and control faeces and urine were collected. Subsequent groups were housed individually in glass metabolism cages on day of dosing. All animals were used in the study, and none were excluded.

Urine and faeces samples were collected on dry ice at 24 h intervals after dosing for up to 96 h. On the day of termination (day four after dosing), the rats were transferred to the post-mortem room. The rats were killed by exposure to a rising concentration of CO_2_. Blood, liver, adipose and kidney samples were collected at termination and stored at −20 °C for further investigation.

All experimental design was performed according to an experimental protocol prepared prior to the commencement of the study.

### 2.5. Measurement of 14C Using Liquid Scintillation Counting (LSC)

All samples directly measured by LSC (dosing solution, urine, faeces, whole blood, and the rest of the tissue samples) were analysed using scintillation “HiSafe” fluid (PerkinElmer Life Sciences, Turku, Finland). All samples were counted using Wallac 1409 DSA Liquid Scintillation Counter (PerkinElmer Life Sciences, Turku, Finland) with WinSpectral software version 2.00.02. All samples were incubated in the dark for at least 30 min to disperse any naturally occurring photoluminescence prior to scintillation counting. Scintillation counting was for 10 min for all samples. The LSC data were corrected for background by subtracting the disintegrations per minute (dpm) value measured from the analysis of a blank sample. For the dosing solution, 5, 10, 20 and 50 µL samples of each dosing solution were added to 4 mL of scintillation “HiSafe” fluid. These were vortexed briefly and allowed to settle for 30 min in the dark before scintillation counting. The remaining dosing solutions were stored at −20 °C.

#### 2.5.1. Analysis of Urine Sample

For urine samples, the total volume of urine collected at each 24 h period was recorded. In total, 50 μL of each urine sample was added to 4mL of scintillation “HiSafe” fluid. These were vortexed briefly and allowed to settle for 30 min in the dark before scintillation counting. The remaining urine samples were stored at −20 °C.

#### 2.5.2. Analysis of Faeces Sample

The total weight of faeces collected at each 24 h period was recorded. The total faecal sample was homogenised, using a Polytron, into a measured volume of phosphate-buffered saline (PBS) to give a 10% homogenate. In total, 500 μL of each homogenate sample was added to 1 mL of “Solvable”, an aqueous-based tissue solubiliser, and incubated in a shaking water bath at 50 °C for 4 h. 500 μL of sodium hypochlorite solution was added to each sample and incubated for a further 30 min. To each homogenised faeces sample, 10 mL of scintillation “HiSafe” fluid was added. These were vortexed briefly and allowed to settle for 30 min in the dark before scintillation counting. The remaining homogenised faeces samples were stored at −20 °C.

#### 2.5.3. Analysis of Blood Samples

A total of 50 μL of each terminal blood sample was added to 4 mL of scintillation “HiSafe” fluid. These were vortexed briefly and allowed to settle for 30 min in the dark before scintillation counting. The remaining terminal blood samples were stored at −20 °C.

#### 2.5.4. Analysis of Tissue Samples

The total weight of each tissue sample collected at termination was recorded. Each tissue sample was homogenised, using a Polytron, into a measured volume of phosphate-buffered saline (PBS) to give a 10% homogenate. A total of 500 μL of each tissue homogenate sample was added to 1 mL of “Solvable” and incubated in a shaking water bath at 50 °C for 4 h. 500 μL of sodium hypochlorite solution was added to each tissue sample and incubated for a further 30 min. To each homogenised tissue sample, 10 mL of scintillation “HiSafe” fluid was added. These were vortexed briefly and allowed to settle for 30 min in the dark before scintillation counting. The remaining homogenised tissue samples were stored at −20 °C.

### 2.6. Data Analysis and Visualisation

Data tables were compiled with the mean and standard deviation (SD) calculated using Excel. The data presented in the summary tables reflect the data recorded in the study records and the data presented in the report text have been rounded appropriately to summarise results. Results visualisation was performed using R (version 4.4.3) and R Studio (version 12.1 + 563) software.

## 3. Results and Discussion

### 3.1. Mass Balance and Excretion

Following administration of 100 mg/kg of higher olefins, all animals appeared healthy with no clinical signs of adversity observed and no influence on the body weight development throughout the study. This outcome is consistent with the low acute oral toxicity studies of higher olefins ranging from C6 to C28, which have reported LD_50_ values of at least 5000 mg/kg across various rat strains (including Fischer 344, Wistar, Sprague-Dawley, and Charles Foster Experimental) [21]. Additionally, previous repeated-dose toxicity studies demonstrated a NOAEL of at least 500 mg/kg bw/day for 1-octene, 1-decene, and 1-hexadecene [4,5].

**Figure 2:**
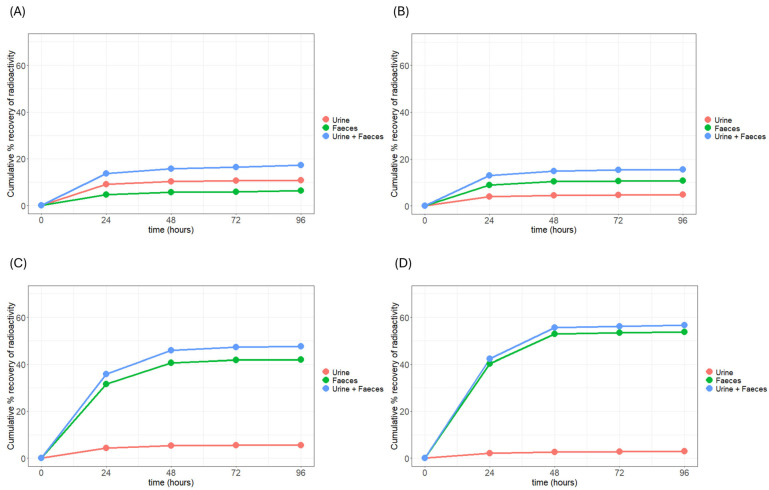
Mean cumulative recovery of ^14^C-derived radioactivity in urine and faeces of rats following an oral administration of 100 mg/kg bw ^14^C-radiolabelled (**A**) 1-Octene, (**B**) 1-Decene, (**C**) 1-Hexadecene, and (**D**) 1-Eicosene.

Approximately 17%, 16%, 48%, and 57% of the administered ^14^C-radiolabelled higher olefins (i.e., 1-octene, 1-decene, 1-hexadecene, and 1-eicosene, respectively) were recovered in excreta within four days post-administration (Figure 2 and Table 2). The extent of ^14^C-derived radioactivity recovered in urine reflects the degree of systemic absorption via the oral route. Cumulative urinary excretion averaged 10.9%, 4.7%, 5.6%, and 2.9% for 1-octene, 1-decene, 1-hexadecene, and 1-eicosene, respectively. A clear inverse relationship was observed between urinary excretion and carbon chain length. This trend is consistent with the previous everted gut sac study, which showed chain-length-dependent intestinal absorption, with the highest absorption rate of 16.2% (expressed as the percentage (%) of total olefin absorbed by Sac per hour) for C8 and less than 1% for C14 and longer chains [1]. Importantly, most urinary radioactivity (approximately 70% and 90% of total ^14^C-derived radioactivity in urine) was eliminated within the first 24 and 48 h, respectively, indicating rapid renal clearance.

In contrast, faeces excretion represented the predominant elimination route for all test compounds except 1-octene. Mean faeces recoveries were 6.4%, 10.8%, 42.0%, and 53.7% of the administered dose for 1-octene, 1-decene, 1-hexadecene, and 1-eicosene, respectively (Figure 2 and Table 2). In opposition to urinary excretion, faeces recovery increased with carbon chain length. Similar to urinary excretion, most faeces radioactivity (approximately 70% and 90% of total ^14^C-derived radioactivity in faeces) was eliminated within the first 24 and 48 h, respectively, indicating rapid elimination.

This observation aligns with Lipinski’s “Rule of Five”, which predicts reduced intestinal absorption for compounds with high lipophilicity (LogP ≥ 5) due to poor solubility in intestinal fluids [14]. As previously reported, the LogP values of higher olefins increase with carbon number, and compounds with nine or more carbons exhibit LogP values > 5 [1]. Therefore, it is reasonable to expect a greater proportion of these compounds to remain unabsorbed and be excreted in faeces. However, the presence of radioactivity in faeces does not rule out the possibility that absorbed olefins were metabolised and subsequently excreted via bile. Further analysis, such as identifying the parent compound in faeces, would be necessary to confirm this.

### 3.2. Distribution of Radioactivity in the Terminal Tissues

**Figure 3:**
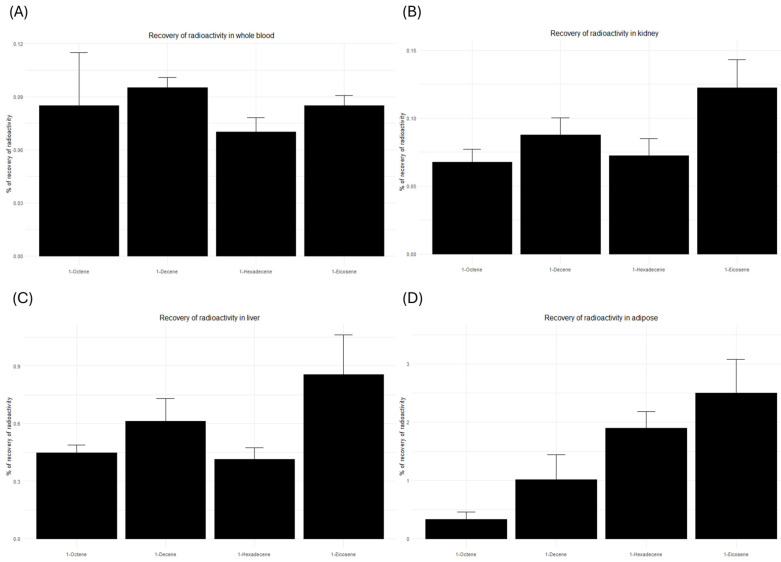
Percentage (mean ± SD) of total dosed ^14^C-derived radioactivity in terminal (**A**) whole blood, (**B**) kidney, (**C**) liver and (**D**) adipose tissue.

The ^14^C-derived radioactivity measured in terminal blood, liver, kidney, and adipose tissues on Day 4 post-dosing is shown in Figure 3 and Table 2. Whole blood levels of ^14^C-derived radioactivity were uniformly low (~0.1%) across all tested higher olefins (Figure 3A). Similarly low ^14^C-derived radioactivity was detected in kidney (~0.1%) and liver (~1%) (Figure 3B,C), although liver levels exhibited a modest increase with chain length.

In contrast, adipose tissue showed a marked increase in retained ^14^C-derived radioactivity with longer carbon chains (Figure 3D). Mean levels in adipose tissue were 0.3%, 1.0%, 1.9%, and 2.5% of total dosed radioactivity for 1-octene, 1-decene, 1-hexadecene, and 1-eicosene, respectively. Notably, 1-eicosene demonstrated approximately an 8-fold higher retention in adipose tissue compared to 1-octene.

Based on previous in vitro findings using everted gut sacs, long-chain olefins such as 1-hexadecene and 1-eicosene are expected to exhibit minimal intestinal absorption. However, minimal absorption does not equate to complete absence of systemic uptake. Even a small absorbed fraction of these highly lipophilic molecules can preferentially partition into adipose tissue once in circulation, leading to measurable retention despite low overall bioavailability. In the present study, the ^14^C-derived radioactivity in adipose tissue increased with chain length (0.3–2.5% of the administered dose), indicating that although the absolute amount absorbed is low, the distribution pattern of the absorbed fraction is strongly influenced by lipophilicity. Importantly, the observed adipose levels should not be interpreted as evidence of extensive systemic absorption, but rather as the expected outcome of selective sequestration of the small absorbed portion into lipid-rich depots. Similar findings were reported by Zahlsen et al. [15], who demonstrated efficient absorption and adipose accumulation of alpha olefins (C8–C10) following inhalation exposure in rats. In that study, concentrations in adipose tissue were 4–20 times higher than in other organs and increased with chain length. Comparable to the present findings, accumulation was greater in the liver than in the kidney, which can be partially explained by the relatively higher lipid content of the liver [14].

### 3.3. Total Recovery of ^14^C-Derived Radioactivity

**Figure 4:**
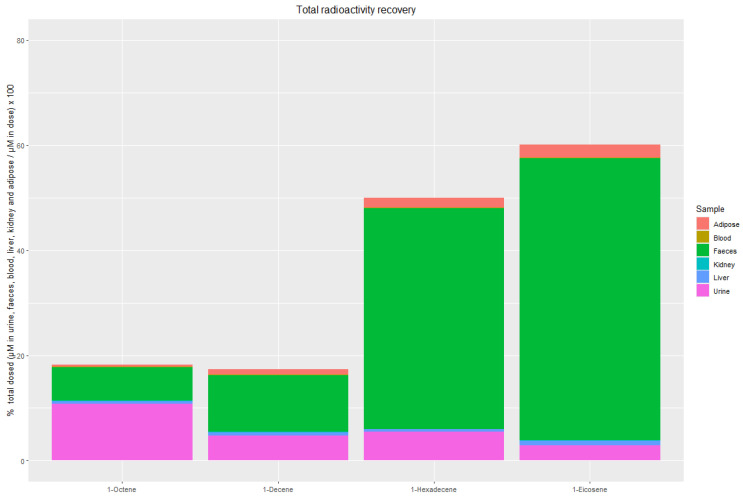
The total recovery of ^14^C-derived radioactivity.

As shown in Figure 4, the total recovery of administered ^14^C-derived radioactivity including excreta and all measured tissues, was relatively low for the shorter-chain olefins but increased with chain length. The total recoveries (expressed as the percentage of the total administered dose) were approximately 18.2%, 17.3%, 50.0%, and 60.1% for 1-octene, 1-decene, 1-hexadecene, and 1-eicosene, respectively.

Within this recovered fraction, faeces accounted for 35.1%, 62.2%, 84.0%, and 89.2% of the total recovered radioactivity, whereas urine contributed 59.8%, 27.3%, 11.1%, and 4.8% of the total recovered radioactivity, for 1-octene, 1-decene, 1-hexadecene, and 1-eicosene, respectively. In contrast, radioactivity measured in adipose tissue, liver, kidney, and blood collectively represented less than 10% of the total recovered fraction, with adipose and liver contributing the highest proportions (<6% and <4%, respectively).

The relatively low overall recovery is not unexpected given the known metabolic fate of higher olefins. The ‘missing’ radioactivity can be explained by three primary mechanisms. First, alpha olefins (or structurally similar fatty acids) can undergo oxidation via cytochrome P450 enzymes during Phase I metabolism, forming alcohols and eventually carboxylic acids [10,22,23]. Carboxylic acid derivatives may be further metabolised through β-oxidation to acetyl-CoA and enter the citric acid cycle, ultimately generating CO_2_, which is exhaled [24]. In the current study, the placement of the ^14^C label at the C4 carbon supports this hypothesis, as β-oxidation cleaves the carbon chain into two-carbon units [25]. Furthermore, evidence suggests that short-chain fatty acids, including short-chain higher olefins, are more readily absorbed and metabolised to CO_2_ compared to their long-chain counterparts, which are more likely to be stored in adipose tissue or metabolised at a slower rate [1,26,27]. This trend aligns with our findings, where greater total radioactivity loss was observed for short-chain higher olefins (e.g., 1-octene and 1-decene) than for long-chain olefins (e.g., 1-hexadecene and 1-eicosene). Since CO_2_ was not captured in the current study, this represents a significant and expected pathway for radioactivity loss, particularly for shorter chains (i.e., 1-octene and 1-decene) which undergo faster systemic turnover.

A second potential contributor to the unrecovered radioactivity is the pulmonary exhalation of unchanged parent olefins. Although the relatively high boiling points and low vapour pressures of 1-octene (121 °C; 17.4 mmHg), 1-decene (171 °C; 1.7 mmHg), 1-hexadecene (285 °C; 2.64 × 10^−3^ mmHg), and 1-eicosene (341 °C; 1.06 × 10^−5^ mmHg) suggest low volatility, their Henry’s Law Constants (HLCs) and low water solubility indicate potential for pulmonary exhalation. Reported HLCs are 0.627, 0.541, 6.1, and 18.9 atm·m^3^/mol for 1-octene, 1-decene, 1-hexadecene, and 1-eicosene, respectively [28]. These values suggest that volatilisation from aqueous alveolar fluid may occur at a rate sufficient to contribute to overall elimination. However, the present study did not include measurement of exhaled air. Therefore, any contribution of parent compound exhalation remains qualitative and hypothetical, and its magnitude cannot be inferred from the current dataset.

In addition to metabolic and potential volatilisation pathways, a portion of the missing radioactivity is likely attributable to technical losses. Given the extreme hydrophobicity of the longer-chain olefins (LogP > 8), adsorption to experimental surfaces (e.g., pipette tips, glass vials, and metabolism cage walls) is a well-recognised source of loss in ADME studies [1,29]. Thus, the reduced mass balance recovery for 1-hexadecene and 1-eicosene is consistent with both their physicochemical properties and the known challenges of handling highly lipophilic materials [29].

### 3.4. Limitation of the Study

While the current study provides valuable comparative data on the disposition of alpha-olefins, several limitations must be acknowledged. First, the total recovery of ^14^C-derived radioactivity was substantially below the threshold for a closed mass balance, particularly for C8 and C10 (17–18%). This is attributed to the lack of experimental apparatus for the collection of exhaled air and the trapping of ^14^CO_2_, which are known elimination pathways for volatile and metabolically active hydrocarbons. Consequently, the “missing” fraction remains unquantified, and the total elimination rate cannot be definitively established. Second, the study did not include metabolic characterisation or identification of specific metabolites in the urine or tissues. All discussions regarding metabolic pathways (e.g., β-oxidation) remain inferential and are based on established literature for analogous alkanes and alkenes rather than direct experimental evidence. Finally, the 96 h observation period represents a standard snapshot of acute disposition, the rapid decline of radioactivity observed in major organs and the low levels remaining at the final time point suggest a favourable clearance profile, though longer-term monitoring would be required to confirm the precise time point of absolute elimination.

## 4. Conclusions

This study provides a comprehensive evaluation of the absorption, distribution, and excretion of selected ^14^C-radiolabelled higher olefins (1-octene, 1-decene, 1-hexadecene, and 1-eicosene) following a single oral dose in male Wistar rats. Excretion data revealed a clear chain-length-dependent trend. Shorter-chain higher olefins (1-octene and 1-decene) showed greater urinary excretion, suggesting higher oral absorption, whereas longer-chain higher olefins (1-hexadecene and 1-eicosene) were primarily eliminated via faeces, indicative of limited intestinal uptake. Tissue retention was minimal in blood, liver, and kidney for all higher olefins as ^14^C-derived radioactivity levels were very low (≤1%) at necropsy. However, low levels of ^14^C-derived radioactivity were still found in adipose tissue, and the amount retained increased with chain length, likely reflecting the greater lipophilicity of longer-chain higher olefins once absorbed. Nevertheless, the overall results show that the elimination of all higher olefins is rapid and ^14^C-derived radioactivity declined significantly within 96 h in the analysed matrices. Total ^14^C-derived radioactivity recovery was incomplete (17–60%), which represents a limitation of the current study design. This missing fraction, particularly notable for shorter-chain olefins, is hypothesised to result from metabolic conversion to ^14^CO_2_ or pulmonary exhalation of the parent compounds. While the observed data provide a valuable baseline for the biological fate of these substances, further research utilising exhaled air sampling and metabolic profiling is required to achieve a closed mass balance and fully confirm the specific metabolic pathways. Overall, these findings highlight how molecular size and lipophilicity govern the initial distribution and excretion of higher olefins, providing relevant data for the regulatory risk assessment of these substances.

## Figures and Tables

**Table 1:** Total radioactivity dosed to each animal.

Group	Rat Number	Dosing Volume Administrated (mL)	Total Radioactivity Dosed (dpm)
1-Octene	1	2.0	4,395,008.0
	2	2.1	4,614,758.4
	3	2.1	4,614,758.4
	4	2.2	4,834,508.8
1-Decene	5	2.3	4,308,652.1
	6	2.3	4,308,652.1
	7	2.4	4,495,984.8
	8	2.3	4,308,652.1
1-Hexadecene	9	2.7	6,173,014.4
	10	2.6	6,173,014.4
	11	2.7	5,952,549.6
	12	2.7	5,952,549.6
1-Eicosene	13	2.8	5,404,959.9
	14	2.8	5,204,776.2
	15	2.7	5,404,959.9
	16	2.7	5,404,959.9

dpm = disintegrations per minute.

**Table 2:** Percentage of total dosed (mean ± S.D.) of each higher olefin after single oral administrated to 100 mg/kg bw/day; n = 4.

Samples	Time	1-Octene	1-Decene	1-Hexadecene	1-Eicosene
Urine	Day 1 (0–24 h)	9.1 ± 3.0	4.0 ± 0.9	4.3 ± 1.1	2.1 ± 1.1
	Day 2 (24–48 h)	1.2 ± 0.4	0.5 ± 0.3	1.0 ± 0.5	0.6 ± 0.3
	Day 3 (48–72 h)	0.3 ± 0.1	0.2 ± 0.1	0.2 ± 0.0	0.2 ± 0.1
	Day 4 (72–96 h)	0.3 ± 0.0	0.1 ± 0.1	0.1 ± 0.0	0.1 ± 0.0
Faeces	Day 1 (0–24 h)	4.7 ± 1.2	8.9 ± 3.9	31.6 ± 9.3	40.2 ± 8.5
	Day 2 (24–48 h)	0.9 ± 0.2	1.4 ± 0.6	9.1 ± 4.5	12.7 ± 1.8
	Day 3 (48–72 h)	0.3 ± 0.1	0.3 ± 0.0	1.2 ± 0.8	0.4 ± 0.4
	Day 4 (72–96 h)	0.5 ± 0.3	0.1 ± 0.1	0.2 ± 0.0	0.3 ± 0.3
Blood	Day 4 (96 h)	0.1 ± 0.0	0.1 ± 0.0	0.1 ± 0.0	0.1 ± 0.0
Liver	Day 4 (96 h)	0.5 ± 0.0	0.6 ± 0.1	0.4 ± 0.1	0.9 ± 0.2
Kidney	Day 4 (96 h)	0.1 ± 0.0	0.1 ± 0.0	0.1 ± 0.0	0.1 ± 0.0
Adipose	Day 4 (96 h)	0.3 ± 0.1	1.0 ± 0.4	1.9 ± 0.3	2.5 ± 0.6

## Data Availability

The data that support the findings of this study are available from the corresponding author upon reasonable request. The data are not publicly available due to HOPA consortia policy.

## Abbreviations

The following abbreviations are used in this manuscript:

ADME	Absorption, Distribution, Metabolism, and Excretion
CAS	Chemical Abstracts Service
COSHH	Control of Substances Hazardous to Health
LD_50_	Lethal Dose 50%
LSC	Liquid Scintillation Counting
NOAEL	No-Observed-Adverse-Effect Levels
PBS	Phosphate-Buffered Saline
SD	Standard Deviation
SOP	Standard Operating Procedures
TK	Toxicokinetic
